# An Epiduroscopy Simulator Based on a Serious Game for Spatial Cognitive Training (EpiduroSIM): User-Centered Design Approach

**DOI:** 10.2196/12678

**Published:** 2019-08-27

**Authors:** Junho Ko, Jong Joo Lee, Seong-Wook Jang, Yeomin Yun, Sungchul Kang, Dong Ah Shin, Yoon Sang Kim

**Affiliations:** 1 BioComputing Lab Institute for Bio-Engineering Application Technology Korea University of Technology and Education Cheonan Republic of Korea; 2 Department of Neurosurgery, Yonsei University College of Medicine Seoul Republic of Korea; 3 Robot Center, Samsung Research Seoul Republic of Korea

**Keywords:** medical education, endoscopy, surgery, catheters

## Abstract

**Background:**

Performing high-level surgeries with endoscopy is challenging, and hence, an efficient surgical training method or system is required. Serious game–based simulators can provide a trainee-centered educational environment unlike traditional teacher-centered education environments since serious games provide a high level of interaction (feedback that induces learning).

**Objective:**

This study aimed to propose an epiduroscopy simulator, EpiduroSIM, based on a serious game for spatial cognitive training.

**Methods:**

EpiduroSIM was designed based on a serious game. For spatial cognitive training, the virtual environment of EpiduroSIM was modeled based on a cognitive map.

**Results:**

EpiduroSIM was developed considering user accessibility to provide various functions. The experiment for the validation of EpiduroSIM focused on psychological fidelity and repetitive training effects. The experiments were conducted by dividing 16 specialists into 2 groups of 8 surgeons. The group was divided into beginner and expert based on their epiduroscopy experience. The psychological fidelity of EpiduroSIM was confirmed through the training results of the expert group rather than the beginner group. In addition, the repetitive training effect of EpiduroSIM was confirmed by improving the training results in the beginner group.

**Conclusions:**

EpiduroSIM may be useful for training beginner surgeons in epiduroscopy.

## Introduction

### Background

An epiduroscopy is a highly effective minimally invasive surgery (MIS) used for chronic lumbago and lumbar disc herniation [1]. In an MIS, a subminiature endoscope (1-mm diameter) and a laser are inserted into a cavity; the endoscope is maneuvered by viewing a 2-dimensional (2D) computed tomography image. Performing high-level surgeries with endoscopy is challenging, and hence, an efficient surgical training method or system is required [2].

Conventional surgical training is performed using surgery observations, animals, cadavers, and plastic models. Surgery observation is achieved by assisting expert surgeons in the operation room. Such surgical training has limited training opportunities, and the interaction between surgical instruments and human organs is difficult to train [3]. Surgical training using animals and cadavers is costly [4], and surgical training using plastic models cannot provide realistic visual and tactile feedback [5].

Recently, surgical training has been performed using a simulator. The simulator provides an opportunity for the trainee to repeatedly attempt various actions, including mistakes, in a virtual environment [6]. In addition, the simulator allows the objective evaluation of the trainee’s training level and can teach rare and dangerous complications at a low cost [4]. As the popularity of video games has increased, serious game technology has been applied to surgical training using a simulator [7]. As serious game provides a high level of interaction (feedback that induces learning) [8], serious game–based simulators can provide a trainee-centered educational environment unlike the traditional teacher-centered education environments [9].

### Objectives

In this study, we proposed an epiduroscopy simulator (EpiduroSIM) based on a serious game for spatial cognitive training. EpiduroSIM aims to provide a trainee-centered educational environment for teaching the insertion paths of the surgical instrument (catheter).

## Methods

### Study Design

In this study, we focused on EpiduroSIM based on a serious game and the cognitive map for self-directed learning of the beginner. Herein, the beginner means a surgeon who has no experience or has low understanding of epiduroscopy. Design of EpiduroSIM and modeling of the virtual environment were achieved through consultation with neurosurgeons.

### Analysis and Design

**Figure 1:**
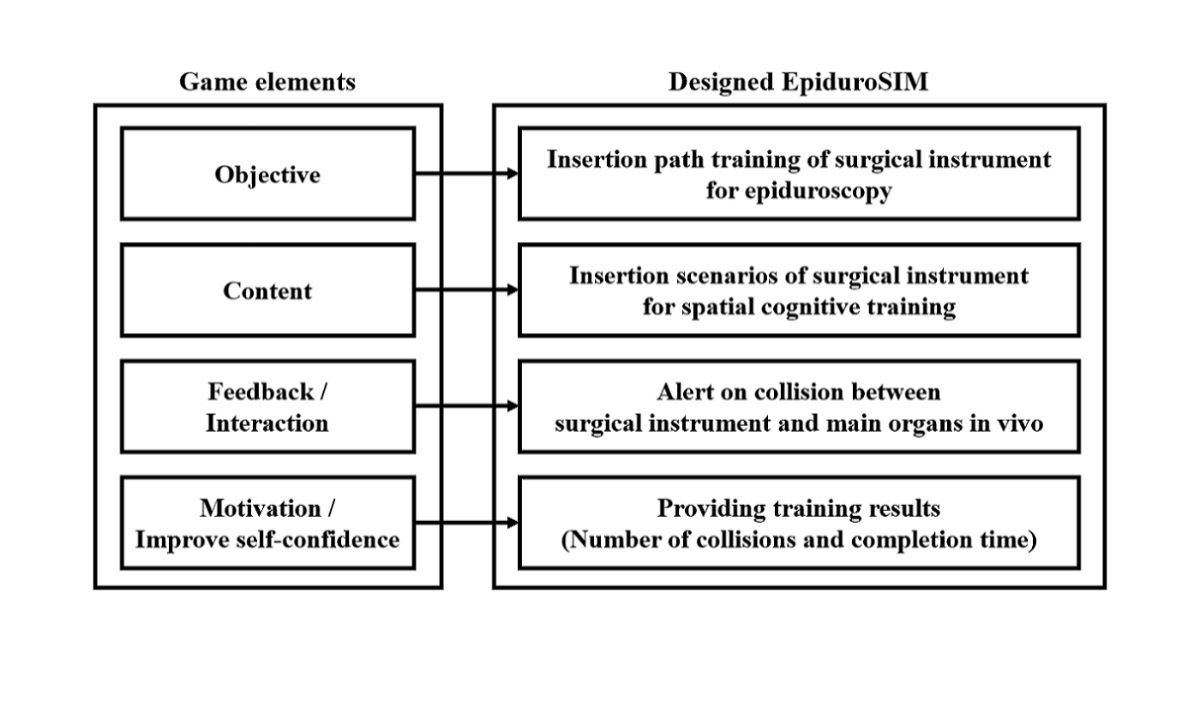
The design of EpiduroSIM based on the game elements of the serious game.

EpiduroSIM was designed based on the game elements of the serious game, as shown in Figure 1. The objective of the designed EpiduroSIM was to teach the insertion paths of the surgical instrument for epiduroscopy, and the content is the insertion scenario of the surgical instrument for spatial cognition training. The feedback and interaction of the designed EpiduroSIM is an alert on the collision between the surgical instrument and the primary organs in vivo. As surgery is generally evaluated based on the operation time (completion time) and mistakes (number of collisions) in surgery [10], the designed EpiduroSIM provides the number of collisions and the completion time as the training results.

Humans form a cognitive map through a series of processes that recognize the space or environment that they experience. In general, the cognitive map consists of an important point called a landmark and a surrounding area connecting these points [11]. This is because it is more effective in human information processing to hierarchically memorize the regions around the important points than to memorize the entire region [12].

The cognitive map is divided into the route map and survey map based on the form and characteristics [13]. The route map means a linear path connecting the primary landmarks between the starting point and destination. The route map is obtained through limited experience with the surrounding environment (if only 1 path is used). People with only the linear path information (route map) are more likely to get lost if they leave the proficient path [14]. The survey map means a map in which the geographical information between the starting point and the destination is configured in a network. The survey map is obtained through various experiences in the surrounding areas and data (maps and aerial photographs) that can illustrate the entire area. People with the survey map are more likely to search for an alternate path if they leave the proficient path. As the experience of the region increases, the route map generally changes to the survey map [15].

**Figure 2:**
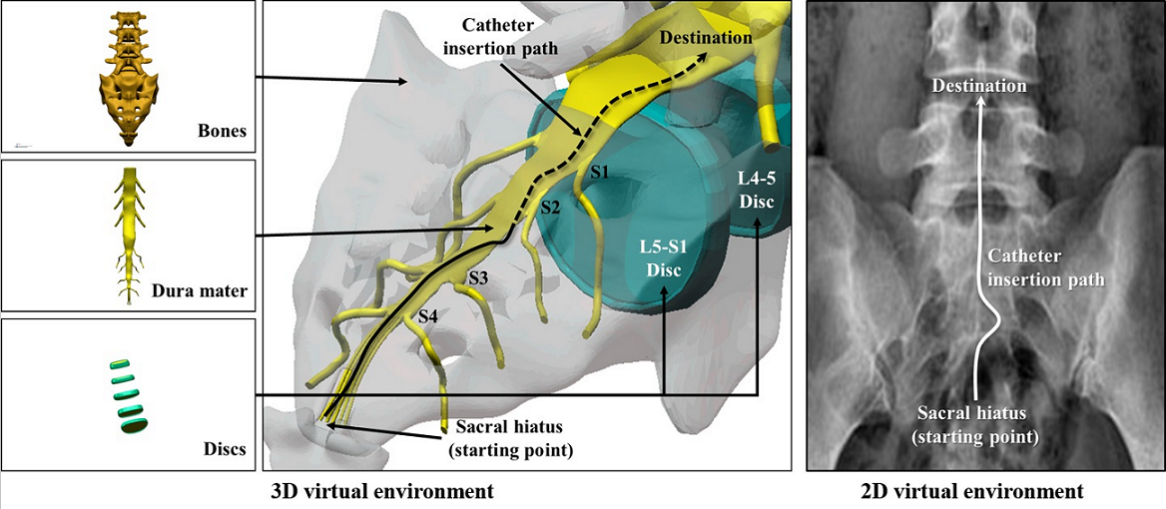
The modeled virtual environments (2D and 3D) based on the cognitive map.

The virtual environment of EpiduroSIM is modeled based on the cognitive map as shown in Figure 2. The modeled virtual environment is divided into a 3-dimensional (3D) virtual environment for the route map and a 2D virtual environment for the survey map. The 3D virtual environment for the route map consists of bones, a dura mater, and discs that serve as landmarks. Through these objects serving as landmarks, the trainee can learn the catheter insertion paths. The 2D virtual environment for the survey map consists of a projected image (fluoroscopy). This allows the trainee to learn the perspective knowledge of all areas of the catheter insertion paths.

**Figure 3:**
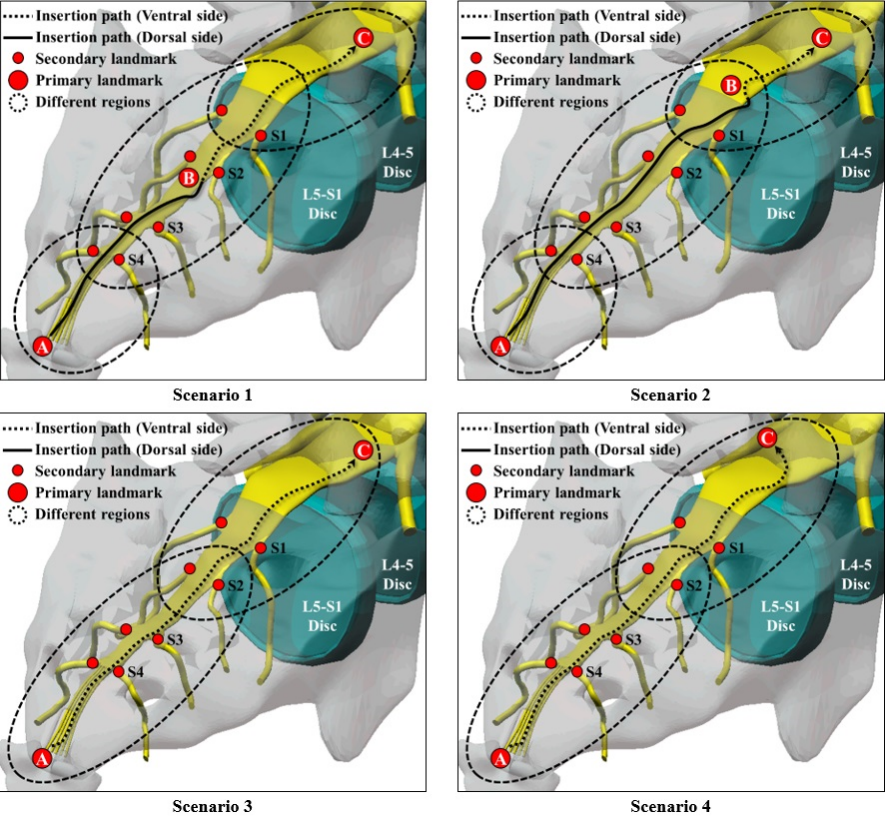
The hierarchical spatial cognitive process in the various catheter insertion scenarios.

Hierarchical spatial cognition [16] does not determine a detailed path (A→S4→S3→B→S2→S1→C in scenario 1 of Figure 3) from the beginning when a person selects a path but selects a primary landmark (A→B→C in scenario 1 of Figure 3) first and subsequently selects a detailed path (A→S4→S3→B→S2→S1→C in scenario 1 of Figure 3). In this path selection process, landmarks with different levels of importance act as partial regions that constitute the whole space. Therefore, hierarchical spatial cognition can be thought as the divide-and-conquer method that resolves a significant problem.

EpiduroSIM provides various catheter insertion scenarios for spatial cognitive training. Figure 3 shows the hierarchical spatial cognitive process in various catheter insertion scenarios. In catheter insertion scenarios in Figure 3, the primary landmark is important for the catheter insertion procedures. In catheter insertion scenarios in Figure 3, the primary landmark A is the starting point where the catheter insertion paths are split into the dorsal side and ventral side in the sacral hiatus. In scenario 1 and scenario 2, the primary landmark B is the point where the catheter insertion paths detour from the dorsal side to the ventral side. In scenario 3 and scenario 4, the primary landmark B is absent because the catheter insertion paths in scenarios 3 and 4 enter the ventral side from the sacral hiatus to reach the lesion disc located on the ventral side without a detour. In catheter insertion scenarios, the primary landmark C is the destination, which is the lesion disc. In catheter insertion scenarios, the secondary landmark is the sacral nerves (S1-4), which is used as a reference point for the trainee to select the detailed paths.

## Results

### Development

**Figure 4:**
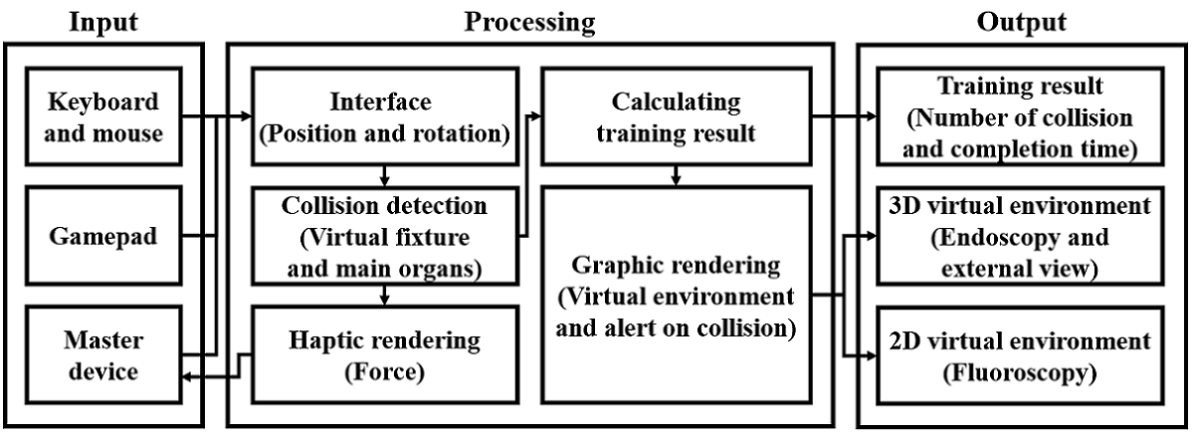
The structure of the proposed EpiduroSIM.

EpiduroSIM consists of the input part, processing part, and output part, as shown in Figure 4. The input part transmits the position and rotation data received from the keyboard or mouse, gamepad [17], and master device [18] to the processing part. The processing part controls the surgical instrument in the virtual environment according to the received position and rotation data. When the surgical instrument collides, it stores the crash record and provides the trainee with visual feedback. Furthermore, the force data calculated at the time of the collision are transmitted to the master device, and the feedback is provided to the trainee. The output part provides visually the virtual environments (Figure 2) and training results in the Database.

**Figure 5:**
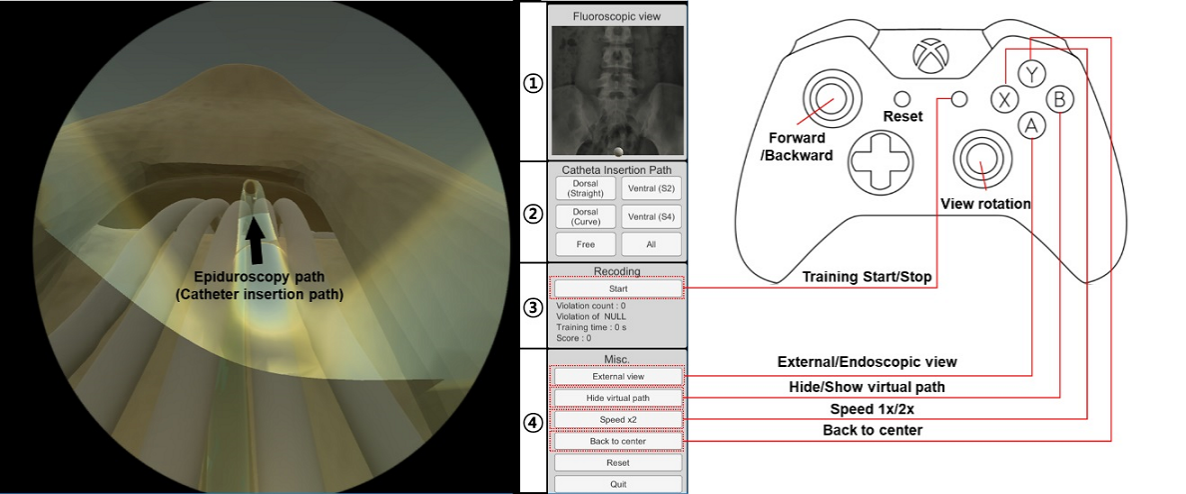
The developed EpiduroSIM considering user accessibility.

EpiduroSIM was developed using Unity3D (Unity technologies Corp), a commercial game engine. The developed EpiduroSIM provides an easy operation method and various functions considering user accessibility, as shown in Figure 5. The functions provided by the developed EpiduroSIM are the fluoroscopic view, the catheter insertion path, the recording, and the miscellaneous menu. The fluoroscopic view function (① in Figure 5) provides a screen similar to the C-arm view used in actual surgery, and the catheter insertion path function (② in Figure 5) provides 4 training scenarios (Figure 3). The recording function (③ in Figure 5) measures and records the training results (number of collisions and completion time) in real time, and the miscellaneous menu function (④ in Figure 5) includes virtual fixture visualization, catheter movement speed control (1x/2x), and position correction at the catheter deviation.

**Figure 6:**
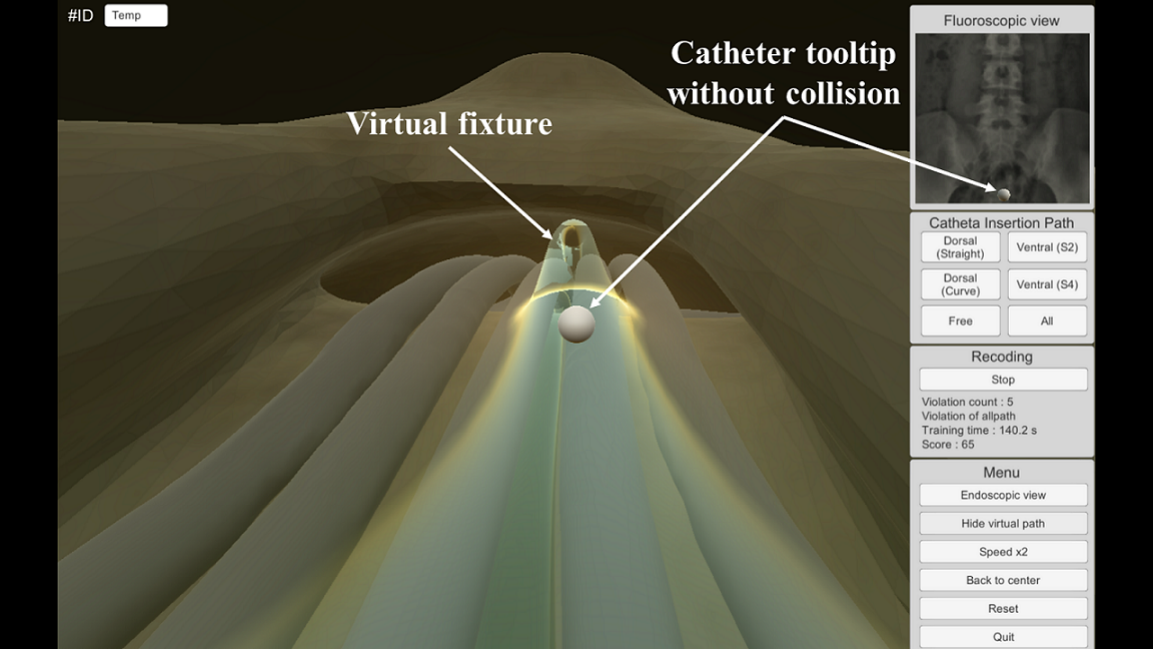
The catheter insertion training without collision.

**Figure 7:**
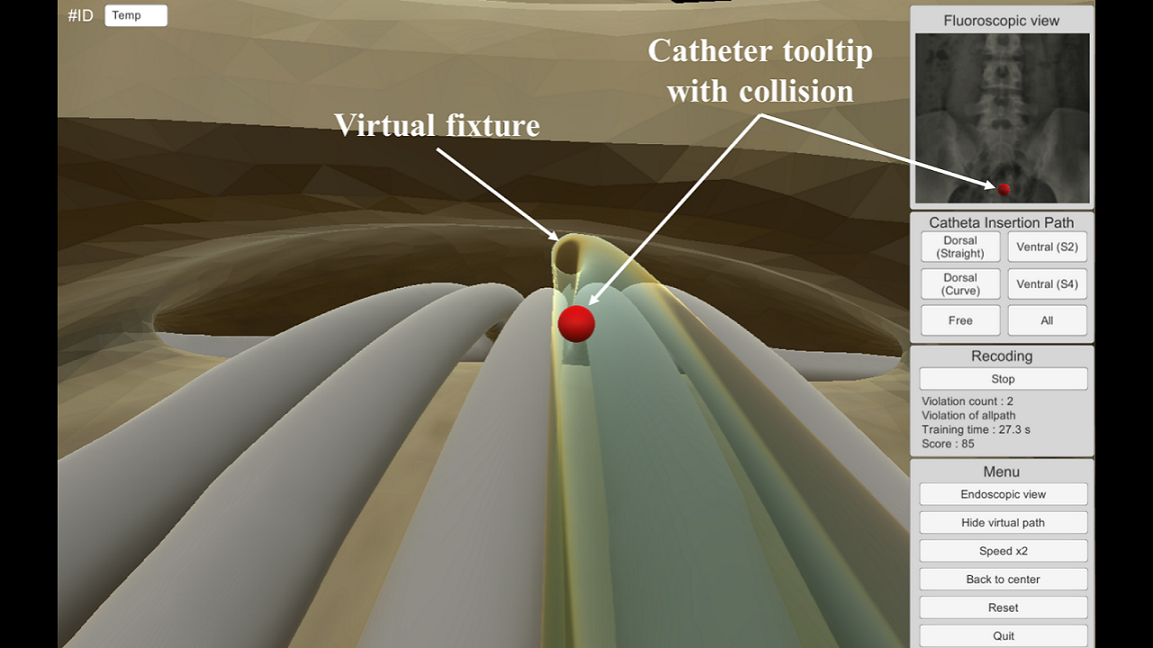
The catheter insertion training with collision.

**Figure 8:**
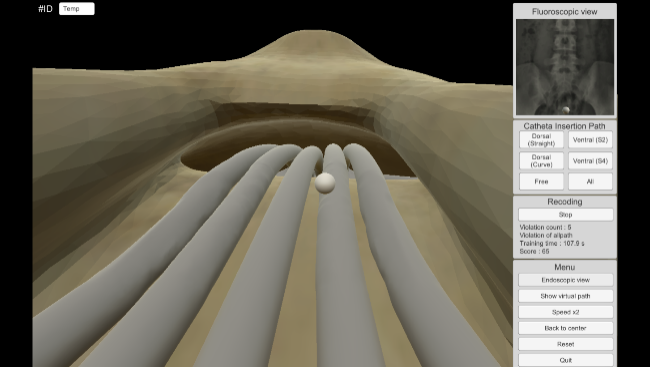
The external view.

**Figure 9:**
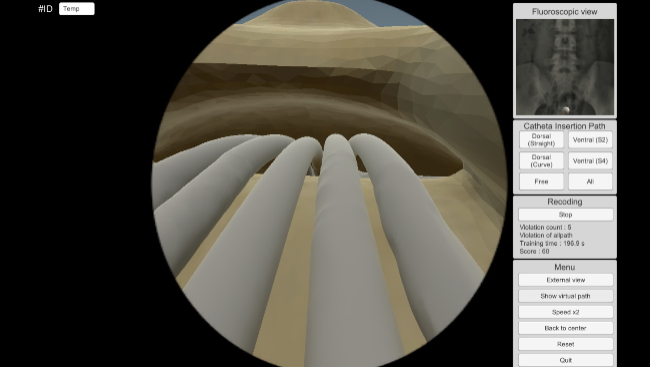
The endoscopic view.

The catheter insertion training in the developed EpiduroSIM proceeds in the following order: (1) select a training scenario, (2) begin training, (3) move the catheter tooltip to the destination (lesion), and (4) validate the training results. If the trainee selects a training scenario other than the free scenario, a virtual fixture in the form of a tube is created, as shown in Figure 6. A virtual fixture is a technique that restricts the motion of a control object to a specified path [19]. The trainee learns the catheter insertion paths along the virtual fixture to the destination. During the training, the catheter tooltip will collide with the virtual fixture if the catheter is out of the insertion path owing to the intentions or mistakes of the trainee. When the catheter tooltip collides, the developed EpiduroSIM records the number of collisions in the training results and provides visual feedback by changing the color (from white to red) of the catheter tooltip, as shown in Figure 7. The trainee can learn the catheter insertion paths in the desired field of view by selecting the external view, as shown in Figure 8, or the endoscopic view, as shown in Figure 9, while moving the catheter tooltip. At the end of the training, the developed EpiduroSIM provides the user with the cumulative number of collisions and completion time.

### Evaluation

The experiments were designed to focus on the fidelity and repetitive training effects. Fidelity represents the degree to which the simulator matches the real environment and skills [20,21]. Fidelity can be divided into physical fidelity and psychological fidelity [22]. Physical fidelity refers to the degree of similarity between a real environment and virtual environment [21,22]. Psychological fidelity refers to the degree to which the skills of real tasks are reflected in the simulator [22]. EpiduroSIM is designed based on a serious game, focusing on psychological fidelity rather than physical fidelity. Therefore, we examined the psychological fidelity of EpiduroSIM in the experiments.

The training result in the simulator based on iterations generally appears as learning curves. This means that the training result is improved as the training using the simulator is repeated. This improvement in the training result does not necessarily imply that the trainee is acquiring actual skills. However, improved training results in simulators that train basic skills such as hand and tool movement can lead to the acquisition of actual skills [23]. This type of verification has been used in many studies as a simulator evaluation tool [24,25]. Therefore, we examined the repetitive training effects of EpiduroSIM in the experiments.

The experiments were conducted by dividing 16 specialists into 2 groups of 8 surgeons. The group was divided into beginner and expert based on their epiduroscopy experience. The sample size (n=16) of the subject was calculated with a .05 significance level, 0.9 power, and 0.3 effect size using G*Power 3 (Heinrich Heine University Düsseldorf) [26].

**Figure 10:**
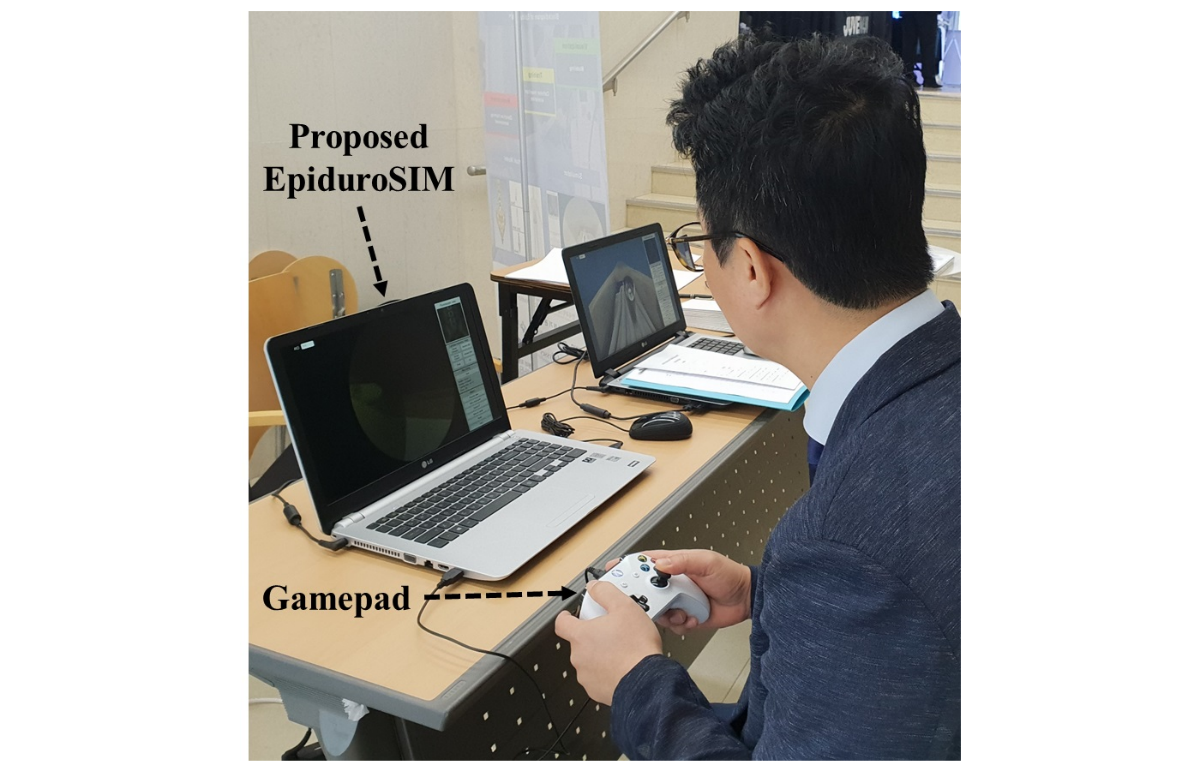
The experimental environment.

The experimental environment consists of EpiduroSIM and gamepad as shown in Figure 10. A gamepad is an input device with higher precision and user preference than a mechanical master device and joystick [27]. In addition, considering a serious game and user accessibility, the gamepad is suitable as the input device of EpiduroSIM. The experiment was conducted through repetitive training (20 times) in the free scenario (an environment without a virtual fixture) of EpiduroSIM. To ensure the reliability of the experimental results, we set the number of repetitions to be higher than the conventional studies [28,29] on the surgical training simulator. The experimental results of repetitive training were analyzed using the repeated-measures analysis of variance. These analyses were performed using SPSS 21 (IBM Corp). A 2-sided significance level of *P*<.05 was set.

**Figure 11:**
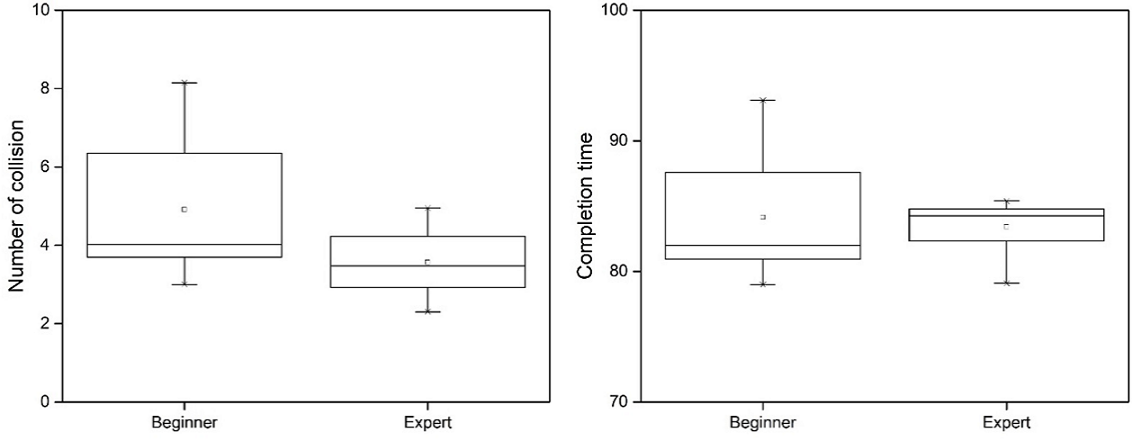
The comparison results (number of collisions and completion time) between the two groups (beginner and expert).

The results of comparison between the 2 groups (beginner and expert) are shown in Figure 11. The mean number of collisions for the beginner group and expert group was 4.91 and 3.56, respectively. The mean completion time of the beginner group and expert group was 84.14 seconds and 83.40 seconds, respectively. The expert group had fewer collisions than the beginner group, and the completion time was faster. This implies that the understanding of actual surgery is well reflected (high psychological fidelity) in EpiduroSIM.

**Figure 12:**
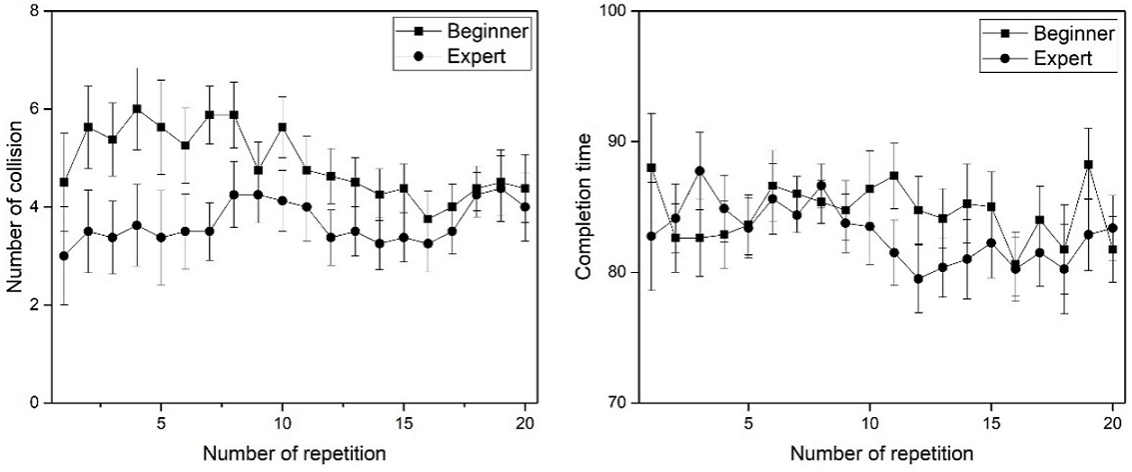
The experimental results (number variation of collision and completion time variation) of repetitive training.

Figure 12 shows the experimental results of repetitive training. As the training was repeated, the beginner group showed a decrease in the number of collisions as a whole and the expert group did not show any significant variation. As the training was repeated, the beginner group had a shorter completion time and the expert group did not show any significant change. From the experimental results, we confirmed that the beginner group exhibited the repetitive training effects, in contrast to the expert group.

## Discussion

### Principal Findings

In this study, we proposed an epiduroscopy simulator (EpiduroSIM) based on a serious game for spatial cognitive training. EpiduroSIM was designed based on a game element of a serious game. The objective of EpiduroSIM was to help beginners learn the insertion path of the catheter inside the human body. Therefore, EpiduroSIM provides various scenarios of catheter insertion to the trainee as content in the virtual environment modeled by the cognitive map. The interaction between EpiduroSIM and the trainee was achieved through visual feedback based on the virtual fixture. Through this interaction, the trainee learns a correct insertion path. Finally, the EpiduroSIM provides the training results of the insertion completion time of the catheter and the mistakes (number of collisions) that occurred during the insertion process to improve the motivation and self-confidence of the trainee.

The experiment for the validation of EpiduroSIM focused on the psychological fidelity and repetitive training effects. On the basis of a comparative result between the expert and beginner groups, it can be concluded that the expert group completed catheter insertion faster with fewer collisions than the beginner group. The result indicates that the understanding of actual surgery is well applied (high psychological fidelity) in EpiduroSIM. From the repeated training results for each group, the beginner group showed a decrease in the number of collisions and completion time. Generally, surgery is evaluated based on the surgical time (completion time) and mistakes (number of collisions) that occur during the surgery. Therefore, the decrease in the completion time and the number of collisions in the beginner group means that the understanding of the surgery has increased. However, there was no repeated training effect in the expert group. The reason could be that experts have a higher understanding of the surgery before training by actual surgical experience compared with the beginners.

### Comparison With Past Work

Cognition is a general term used to describe mental activities related to thinking, learning, and memory [30]. Cognitive training is applied in the form of mental imagery without actual physical movements [31]. Cognitive training has been used in sports and has become an important tool used by athletes at the elite level [32]. In addition, cognitive training has been applied in the form of simulators in the aeronautical field to improve flight performance through pilot training [33].

Owing to the proven efficacy in the sport and aviation sectors, studies have been conducted on using cognitive training for surgical training. In a study on the relationship between surgical proficiency and cognitive processes, surgical competence reportedly combined the intellectual movements of decision making with the ability to perform mechanical tasks [34]. In a study involving 58 expert surgeons, 70% agreed that cognitive skill was one of the best traits required by the trainee surgeons [35].

Owing to MIS, the ability to perform mechanical tasks has become important but the importance of cognitive skills has not diminished. As laparoscopic surgeries and robotic surgeries require significant levels of cognitive and decision-making abilities [24], a few simulators have been studied to provide cognitive training to surgeons [7,23,36]. In these studies, a variety of virtual reality tasks were designed to improve specific cognitive skills such as movement planning, working memory, and preparatory attention. In other studies, error recognition and the cognitive simulator feedback were evaluated [37,38]. Although these cognitive simulators have been developed focusing on surgical procedures, spatial cognitive training for learning the insertion paths of surgical instruments in the human body was not considered.

We herein proposed EpiduroSIM based on a serious game for spatial cognitive training. To learn the catheter insertion paths in the human body, EpiduroSIM was designed based on the game elements of the serious educational game. For spatial cognitive training, the virtual environment of EpiduroSIM was modeled based on a cognitive map. EpiduroSIM was developed to provide various functions considering user accessibility. The experiment for the validation of EpiduroSIM focused on the psychological fidelity and repetitive training effects. The psychological fidelity of EpiduroSIM was confirmed through the training results of the expert group rather than the beginner group. In addition, the repetitive training effect of EpiduroSIM was confirmed by improving the training results in the beginner group.

### Limitations

Serious games based on the game elements for entertainment are differentiated from simulators for reflecting reality [39]. A serious game with game elements such as competition, self-confidence, environment, objectives, and rules has the advantage of improving the self-direction, personality, and persistence of learning compared with the simulator [40]. Despite the advantages of a serious game, the following 7 areas of study are required for the effective development of a serious game for medical education [41]: (1) disposition to engage in learning, (2) impact of realism and fidelity on learning, (3) threshold for learning, (4) process of cognitive development during knowledge gain, (5) stability of knowledge gain (retention), (6) capacity for knowledge transfer to related problems, and (7) disposition toward sensible action within clinical settings. Among these areas, our study focused on the process of cognitive development during knowledge gain.

Therefore, EpiduroSIM provides high-level accessibility to beginners for self-directed learning without past knowledge of the simulator, but it has a limitation that it does not provide the same experience as actual surgery such as the kinesthetic sense.

### Conclusions

Serious games have begun to be applied to various applications, including medical education. However, further studies are still required to popularize serious games. We herein applied the cognitive map to the insertion paths training of a surgical instrument to study the cognitive development process during knowledge gain. In the future, we expect the importance of cognitive training in surgical education to increase, and we hope that the results of this study will be used in cognitive training studies.

## Abbreviations

2D: 2-dimensional
3D: 3-dimensional
MIS: minimally invasive surgery
